# Comparison between inflammatory biomarkers procalcitonin, IL-6 and C-reactive protein for infection diagnosis and fever evolution in neutropenic patients, submitted to hematopoietic stem cell transplantation

**DOI:** 10.1186/cc12927

**Published:** 2013-11-05

**Authors:** Karin Schmidt Rodrigues Massaro, Rodrigo Macedo, Maria Aparecida Shikanai-Yasuda, Silvia Figueiredo Costa

**Affiliations:** 1 Department of the School of Medicine of Universidade de São Paulo, Brazil; 2 Fanem Ltd, Brazil, São Paulo, Brazil; 3 Infectious and Parasitology Diseases, Department of the School of Medicine of Universidade de São Paulo, Brazil

## Background

Biomarkers were assessed during neutropenic fever in hematopoietic stem cell transplantation (HSCT). The objective was to assess serum values of C-reactive protein (CRP), procalcitonin (PCT) and IL-6 to identify infection in HSCT and risk factors for death.

## Materials and methods

Prospective study with 296 patients submitted to autologous or allogeneic HSCT. PCT, CRP and IL-6 dosed at the following moments: afebrile neutropenia, fever, 24 hours upon fever, 72 hours upon fever and long-lasting fever. Patients were classified into groups (I, afebrile; II, fever of unknown origin; and III, clinically or microbiologically proven fever). ROC curves, sensitivity, specificity, and multivariate analysis were used to evaluate factors associated with death.

## Results

One hundred and ninety patients had fever. Mean and median values of IL-6 at fever onset in group I with regard to group II (*P *= 0.013) presented significantly higher values. Levels of CRP in group I differed significantly from those found in group III (*P *< 0.05). Groups differed in levels of IL-6 and CRP at fever onset. Group II presented IL-6 and CRP concentrations significantly lower than group III. Cutoff values of PCT: fever onset, 24 hours upon fever, 72 hours of fever, and long-standing fever were: 0.32; 0.47; 0.46 and 0.35 µg/l. At fever onset, sensitivity was 52.3 and specificity 52.6 for infection diagnosis. Best cutoff values of CRP for fever onset, 24 hours upon fever, 72 hours upon fever and long-standing fever were: 79, 120, 108 and 72 mg/l. At fever onset, sensitivity was 55.4 and specificity was 55.1. Best cutoff values of IL-6 for fever onset, 24 hours upon fever, 72 hours upon fever and long-standing fever were: 34, 32, 16 and 9 pg/ml. At fever onset, sensitivity and specificity were: 59.8 and 59.7. In the autologous' group, IL-6 presents significant values at initial moments. Independent risk factors identified in the multivariate analysis were: related donor, unrelated donor, Gram-negative infection, DHL ≥390 (UI/l), urea ≥25 (mg/dl) and CRP ≥120 (mg/l).

## Conclusions

IL-6 and CRP are associated with the early diagnosis of clinically or microbiologically confirmed infection in post-HSCT febrile neutropenia. The association of the three biomarkers did not present any advantage, nor did it improve diagnostic accuracy. IL-6 was the only biomarker significantly associated at an early stage with infection when assessed only in patients submitted to autologous HSCT. The independent variables associated with death were: allogeneic transplantation, Gram-negative infection, DHL ≥390 UI/l at fever onset and urea ≥25 mg/dl at fever onset and CRP ≥120 mg/l.

